# Treatment of Cesarean Scar Pregnancy with Traditional Chinese Medicine and Warming Moxibustion

**DOI:** 10.1155/2022/5835569

**Published:** 2022-05-17

**Authors:** Xiaqin Cai, Hengxing Shi, Senge Dai, Jiajia Wang, Bin Lu, Qing Zhang

**Affiliations:** Tongde Hospital of Zhejiang Province, Hangzhou City, Zhejiang Province, China

## Abstract

We aimed to report the clinical characteristics of cesarean scar pregnancy (CSP), improve the understanding of uterine scar pregnancy, and assess the outcomes of our treatment strategy for CSP. We present 30 patients with CSP diagnosed by transvaginal ultrasonography. Patients received B ultrasound-guided lauromacrogol injection, followed by evacuation under B ultrasound guidance, and intrauterine balloon compression for hemostasis. Postoperatively, all patients received Bushenquyu decoction and warming moxibustion. All patients showed fast recovery and preserved fertility. The combination of lauromacrogol injection and suction curettage under hysteroscopic guidance is an effective conservative treatment for CSP that can help preserve the reproductive function. Postoperative traditional Chinese medicine and warming moxibustion may reduce the risk of ectopic pregnancy and increase the rate of subsequent intrauterine pregnancy.

## 1. Introduction

Cesarean scar pregnancy (CSP) refers to ectopic implantation of the embryo in the uterine scar. The estimated incidence of CSP ranges from one case per 1800 to one case per 2200 pregnancies [1]. Previous history of a cesarean section is the main risk factor for CSP. Other risk factors include traumatic curettage, manual evacuation of placenta, history of myomectomy, metroplasty, and assisted reproductive techniques. According to a recent study, the cesarean delivery rate in China has increased from 28.8% (3788029/13160634) in 2008 to 36.7% (4 997685/13608933) in 2018 [2]. This has contributed to a considerable increase in the reported incidence of CSP over the last decade. Pregnancy on the lower segment cesarean section scar is a rare but catastrophic complication with a high incidence of fetal and maternal morbidity [3]. In cases where the embryo sac is implanted at the scar, the villi progressively invade the myometrium with the progression of pregnancy. In severe cases, it can penetrate through the uterus causing uterine rupture requiring hysterectomy [4]. The clinical presentation mainly includes vaginal bleeding and lower abdominal pain, which mimic the clinical manifestations of threatened abortion, tubal pregnancy, and other diseases. If CSP is misdiagnosed as early pregnancy, abortion can lead to potentially fatal intraoperative bleeding that may necessitate hysterectomy [5]. The diagnosis of CSP usually depends on auxiliary examinations, especially the transvaginal Doppler ultrasound and other imaging examinations.

Despite the increased awareness of CSP in recent years, management options have been primarily influenced by published case reports rather than consensus or evidence-based guidelines. Currently sued options for management of CSP include systemic and/or local injection of methotrexate, local injection of lauromacrogol, uterine curettage, laparoscopic resection and repair of the implantation site, uterine artery embolization, or hysterectomy. The treatment is generally tailored to the patient based on gestational age, clinical presentation, and the existence of fetal heart activity, and it differs between practitioners and clinics [6–8]. Furthermore, some treatments may cause side effects (such as hepatorenal toxicity) and lead to infertility.

In China and East Asia, traditional Chinese medicine (TCM), mainly including herbal medicine, acupuncture, and moxibustion, is frequently used for the treatment of gynecological diseases [9]. In a study of 8766 Taiwanese infertile women, 96.17% were found to have used Chinese herbal products in addition to routine gynecological therapies [10]. Moxibustion is also commonly used to treat gynecological diseases for its functions of warming meridians and dispersing cold, strengthening the body, and eliminating diseases. Nevertheless, there is a paucity of published evidence to support the TCM treatment. Therefore, we reviewed the diagnosis, management, and treatment outcomes of 30 women with early live CSP who received ultrasound-guided lauromacrogol injection, uterine evacuation, and Foley balloon compression hemostasis, combined with traditional Chinese medicines and warming moxibustion between March 2018 and August 2021 at our center.

## 2. Materials and Methods

### 2.1. Patients

**Figure 1:**
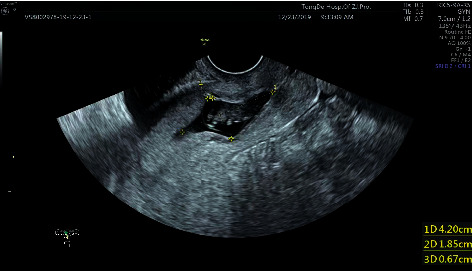
B ultrasound acoustic image of a 38-year-old CSP patient who presented with amenorrhea for 59 days and vaginal bleeding along with low back pain for 10 days. The gestational sac was located on the scar on the anterior wall of uterine isthmus with pulse of the primitive heart tube. The size of the gestational sac is 4.2 × 1.9 × 2.8 cm.

Medical records of 30 patients with early live CSP who were admitted to the Tongde Hospital of Zhejiang Province between January 2018 and August 2021 were retrospectively analyzed. The mean age of patients was 33 ± 5 years (range, 19–44); the mean *β*-HCG level was 47008.3 ± 42992.9 mIU/mL (range, 2218.2–130618.9); the mean gestational age was 48 ± 8 days (range, 33–70); and the length of hospital stay ranged from 2 to 4 days. All patients underwent transvaginal ultrasound. The sonographic criteria for diagnosis of CSP were as follows: (1) abnormal uterine incision scar with an empty cervical canal and uterine cavity; (2) gestational sac or mass situated at the anterior uterine isthmus and thinned or absent myometrium between the gestational sac and bladder; (3) gestational sac within the anterior portion of the lower uterine segment at the presumed site of the cesarean scar; and (4) detection of circular blood flow signal around the gestational sac or mass by the Doppler color flow imaging [11, 12] (Figure 1). History of amenorrhea, increased serum *β*-HCG level, history of a cesarean section were also considered, in addition to ultrasound examination findings [13].

Among the 30 patients, 4 (13.3%) patients had lower abdominal pain, 14 (46.7%) had vaginal bleeding, 4 (13.3%) had vaginal bleeding with lower abdominal pain, 13 (43.3%) had no symptoms, and 1 had bleeding after legal abortion at a local hospital. Two patients had a history of 2 previous CSP, and 1 patient had a history of 3 previous CSP.

The study was approved by the Ethics Committee of the Tongde Hospital of Zhejiang Province. Informed consent was obtained from all participants.

## 3. Materials and Methods

### 3.1. Routine Treatment

The results of routine blood and urine tests, liver and kidney function tests, coagulation profile, and ECG were normal in all 30 patients. Prior to the procedure, preparations for blood transfusion and resuscitation were made. None of the patients had any surgical contraindications.

All procedures were performed in the lithotomy position. Under aseptic conditions, the tip of a 9-gauge needle was inserted under ultrasound guidance after determining the location of the gestational sac. After sucking fluid from the gestational sac, 10 mL lauromacrogol was slowly injected at several locations surrounding the peritrophoblastic tissue. The gestational sac shrunk with patchy or ring-like enhancement, and little amount of blood flow was observed peripherally with the transvaginal color Doppler flow imaging. After the operation, the patient returned to the ward to rest. In one patient who experienced excessive vaginal bleeding due to transfer from another hospital, bilateral uterine artery embolization was performed after injection of lauromacrogol. Aspiration was performed under abdominal ultrasound guidance after 24 h. First, cervical dilatation was carefully and successively accomplished. Under B ultrasonography monitoring, a suction tube was inserted directly in the gestational sac for curettage and suction with a negative pressure to empty the uterine cavity. After suctioning out the gestational sac, 10 U Pitocin was injected into the posterior uterine neck, immediately followed by intrauterine balloon compression to achieve hemostasis. No. 20–24 Foley dual lumen catheters were used depending on the amount of intraoperative blood loss. The volume of the blood loss was measured after the drained blood was collected from the drainage bag attached to the other end of the catheter. Half of the fluid within the balloon was extracted after 6 h of balloon compression, if there was only a small amount of pink-colored liquid in the drainage bag. The balloon withdrawal process was performed on the morning of the second day. Patients with no obvious vaginal bleeding and apparently decreased HCG were discharged.

### 3.2. TCM Treatment

All patients were treated with Bushenquyu decoction and warming moxibustion on the first day after surgery. The Bushenquyu decoction is a formula consisting of *Astragalus membranaceus*, *Atractylodes macrocephala*, *Codonopsis pilosula*, *Angelica sinensis*, *Ligusticum chuanxiong*, motherwort, peach kernel, dodder, *Eucommia ulmoides*, Rehmannia, tortoise plastron, baked ginger, *Morinda officinalis*, and *Pyrola calliantha*. The decoction has the effect of invigorating Qi and blood, promoting blood circulation, and alleviating blood stasis. One dose of decoction was administered on each day for 21 days.

**Figure 2:**
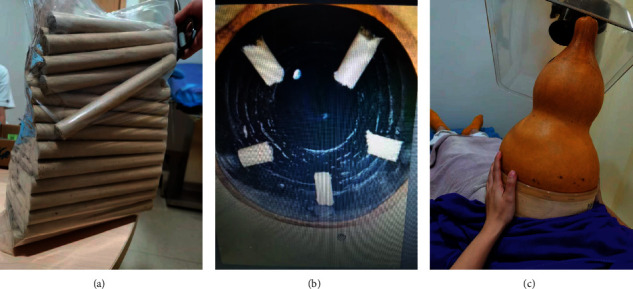
Treatment of cesarean scar pregnancy with medicinal warming moxibustion. (a) Moxibustion. (b) Collar base. (c) Nanocloth cover.

The warming moxibustion treatment mainly consists of *Radix Linderae*, *Evodia rutaecarpa*, fenugreek, *Achyranthes bidentata*, *Caulis spatholobi*, *Radix Saposhnikoviae*, and refined moxa. It dispels cold to relieve pain, promotes blood circulation, and alleviates blood stasis. A warming moxibustion apparatus of dimensions 0.5 cm in diameter and 4–5 cm in length was used. The patients were placed in the supine position and the lower abdominal skin was exposed. The physician applied the essential oil evenly to the abdomen, and massaged on the selected acupoints including Shenque (CV 8), Tianshu (ST 25, bilateral), Zigong (CV 19), Qihai (CV 6), and Guanyuan (CV 4). Then, the physicians placed the collar base with a filter screen on the patient's lower abdomen. The collar base holds 5 moxibustions and was covered with a warming moxibustion apparatus made by gourd (Figure 2). The warming moxibustion apparatus was further covered with a nanocloth to reduce heat loss. Warming moxibustion was performed 2–3 times a week and one course comprised of 6–10 treatments. After recovery of menstruation, the second course of treatment was conducted, and the continuous 3-month period was considered as single treatment period. Patients were required to use adequate contraception during treatment.

### 3.3. Statistics

Data analysis was performed using SAS 9.4 (SAS Institute, North Carolina, US). Data for continuous variables are presented by the mean ± standard deviation (SD). For categorical variables, the number of patients identified and by percent is presented.

## 4. Results

**Figure 3:**
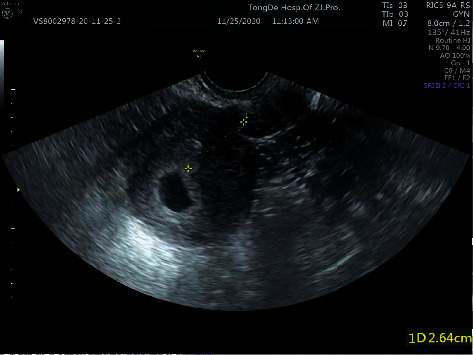
B ultrasound acoustic image of same patient in Figure 1 showing normal intrauterine pregnancy after treatment. The gestational sac is located in the uterus (size: 1.9 × 1.4 × 1.5 cm). The germ and the pulse of the primitive heart tube can be observed.

After the surgery, all 30 patients exhibited a small amount of vaginal bleeding. The mean HCG level was 14226.6 ± 12673.6 mIU/mL (range, 370.7–42997.0). The average amount of bleeding during curettage was 42 ± 33 mL (range, 10–150). All surgeries were completed in a single sitting with no incidence of heavy vaginal bleeding or other complications. Postoperative pathological examination of tissues revealed chorionic villi and decidua. At 15-day follow-up, the average *β*-HCG level was 52.5 ± 46.3 mIU/mL (range 1.2–155.8), and all patients showed normal blood routine and liver function tests and normal acoustic images on B ultrasound. All patients regained the normal menstrual cycle on subsequent follow-up. Three patients used an intrauterine device or other contraceptive methods; 6 patients were lost to follow-up. Twenty-one patients had normal intrauterine pregnancy after 6 months (Figure 3). Among these 21 patients, 9 patients had abortion and 12 patients had normal pregnancy lasting more than 11–12 weeks. Three patients had intrauterine contraceptive rings and other contraceptive methods.

## 5. Discussion

In this study, we retrospectively assessed the outcomes of our management strategy for CSP in a cohort of 30 patients treated at our center. All patients received ultrasound-guided lauromacrogol injection, followed by uterine evacuation and intrauterine balloon compression to achieve hemostasis. After the operation, all patients received herbal medicines and warming moxibustion and showed satisfactory outcomes.

Owing to advances in medical science, most cases of CSP can be diagnosed by transvaginal ultrasonography in the early stage; however, the treatment methods for CSP remain divergent [14, 15]. Improper management of CSP can cause massive bleeding necessitating repeated manual evacuation and hysterectomy and can lead to infertility and even loss of life. Treatment methods have changed from simple total hysterectomy to conservative management using multiple methods. Killing of an embryo with mifepristone plus methotrexate plus curettage under the guidance of B ultrasound can lead to prolonged hospital stay; in addition, methotrexate has several side effects such as oral ulceration, elevated liver enzymes, bone marrow depression, and bleeding [16]. Hysteroscopic electrotomy can cause damage to the uterine muscle fibers, causing uterine perforation, abdominal pain, and uterine cavity adhesion, resulting in amenorrhea. Uterine artery embolization (UAE) is mainly used to prevent bleeding prior to the removal of the gestational sac at the scar site of the uterus and to achieve emergency hemostasis in case of massive hemorrhage. However, UAE can lead to embolism to other body parts after operation, abdominal pain caused by uterine ischemia, intrauterine adhesions caused by endometrial ischemic necrosis, fever, and allergy [17]. Hysteroscopy has the greatest advantage of visualization and can be used for the treatment of CSP; however, studies have recommended that the thickness of the myometrial layer in a cesarean scar should be more than 3 mm [18].

The treatment strategy utilized at our center can overcome the drawbacks of existing CSP treatment techniques. Lauromacrogol, also known as polyoxyethylene 10 lauryl ether, is a sclerosing agent commonly used for sclerotherapy of varicose veins in the lower extremities, esophageal and gastric varices, pancreatic pseudocysts, uterine myomas, and all kinds of hemangiomas [19]. The injection of lauromacrogol into the perivascular tissues can cause fibrosis, resulting in vascular compression and hemostasis [20]. The drug does not travel through the uterine artery and vein, distant blood vessels, or ovarian blood vessels, which helps avoid uterine and ovarian damage caused by reduced blood supply [21]. Compared with curettage after UAE, vacuum aspiration after ultrasound-guided local lauromacrogol injection offers the advantages of minimal bleeding, short hospitalization duration, rapid decline in *β-*HCG level, and very few adverse reactions.

According to the traditional Chinese medicine theory, patients with postpartum diseases often have deficiency and stasis. Surgical management for CSP damages not only the uterus but also vital energy, Qi, and blood. Damaged vital energy and Qi deficiency will impair blood circulation and cause stasis. Simultaneously, the leakage of blood through the damaged uterine vessels causes weakness inside vessels and stasis outside vessels. Exposure to wind, cold, and dampness will further induce blood stasis. Therefore, we use self-designed Bushenhuoxue decoction to invigorate kidney Qi, promote blood circulation, and alleviate blood stasis. *Astragalus membranaceus*, *Codonopsis pilosula*, and *Atractylodes macrocephala* replenish Qi to invigorate the spleen. *Angelica* can replenish blood, nourish blood, activate blood, regulate menstruation, and stop bleeding and have a better effect with herbs nourishing kidney. *Ligusticum wallichii* moves Qi in blood. Motherwort and peach kernel promote blood circulation, remove blood stasis, and regulate menstruation. *Semen Cuscutae*, *Eucommia ulmoides*, *Rehmannia* nourish yin, kidney, and liver. *Tortoise plastron* nourishes yin and heart blood, regulates conception and thoroughfare vessels, and alleviates blood stasis. Baked ginger warms vessels and promotes blood circulation. *Morinda officinalis* and *Pyrola calliantha* warm the kidney to strengthen yang, nourish liver and kidney, and strengthen bones. Many Chinese herbs have been shown to exhibit estrogen-like effects by stimulating biosynthesis of estrogen, increasing endometrial thickness, and enhancing the expression of estrogen receptors by altering the molecular pathways and gene expression [22, 23]. Blood-activating drugs increase arterial blood flow, reduce vascular resistance, improve blood circulation, so as to increase uterine blood supply, improve internal environment, and promote scar repair [24].

Warming moxibustion has been shown to be effective in the treatment of premature ovarian failure, infertility, dysmenorrhea, and other gynecological diseases [25, 26]. Some pharmacological studies have found that the active ingredients in *Artemisia* can promote blood circulation and increase blood flow to ovaries, thus further improving the chances of ovulation and pregnancy [27, 28]. We used warming moxibustion combined with *Lindera aggregata*, evodia, and fenugreek to warm channel and remove coldness; *Achyranthes bidentata,* to promote blood circulation and alleviate stasis, nourish kidney and liver, and strengthen bones; *Sargentodoxa cuneata,* to clear away heat and toxic substances and promote blood circulation; and *Saposhnikovia divaricate,* to clear heat for calming endogenous wind. Moxa wool is the raw material of moxibustion strips, it also has a hemostatic effect, dispels cold, removes dampness, and eliminates fatigue. We further use natural gourd to establish a semiclosed state that reduces evaporation and diffusion; the consequent increase in pressure helps increase the drug concentration, creating a high concentration gradient, delivering the drug across the skin and into the systemic circulation. It directly acts on the disease site by dilating blood vessels, promoting local blood and lymphatic circulation, and improving the nutritional status in specific tissues [29]. Medicinal moxibustion directly enters the three yin meridians and goes straight down the coke. With the power of pure yang moxibustion, it can ignite the fire into the vital energy.

This study has certain limitations. First, the patients were enrolled at a single center with small sample size, and the follow-up period was not long enough. Further studies are required prior to recommending our treatment regimen for individuals with early live CSP. Furthermore, this study lacked a control arm of CSP cases who received routine treatment. The treatment strategies for other stages and types of CSP are still being studied.

## 6. Conclusion

Patients with early live CSP can benefit from a management strategy using a combination of B ultrasound-guided lauromacrogol injection, uterine evacuation, intrauterine balloon compression hemostasis, and Bushenhuoxue decoction with warming moxibustion. This treatment strategy can facilitate restoration of menstrual cycles, help preserve the reproductive function, and prevent the incidence of CSP during the next gestation.

## Data Availability

The datasets generated and analyzed during the present study are available from the corresponding author on reasonable request.

## References

[1] Seow K.-M., Hwang J.-L., Tsai Y.-L., Huang L.-W., Lin Y.-H., Hsieh B.-C. (2004). Subsequent pregnancy outcome after conservative treatment of a previous cesarean scar pregnancy. *Acta Obstetricia et Gynecologica Scandinavica*.

[2] Li H.-t., Hellerstein S., Zhou Y.-b., Liu J.-m., Blustein J. (2020). Trends in cesarean delivery rates in China, 2008-2018. *JAMA*.

[3] Liu Y., Zhao Y. (2016). Early diagnosis and treatment of scar pregnancy after cesarean section. *Clinical Research*.

[4] Guise J.-M., McDonagh M. S., Osterweil P., Nygren P., Chan B. K. S., Helfand M. (2004). Systematic review of the incidence and consequences of uterine rupture in women with previous caesarean section. *BMJ (Clinical research ed)*.

[5] Jiao L.-z., Zhao J., Wan X.-r. (2008). Diagnosis and treatment of cesarean scar pregnancy. *Chinese Medical Sciences Journal*.

[6] Litwicka K., Greco E. (2013). Caesarean scar pregnancy: a review of management options. *Current Opinion in Obstetrics and Gynecology*.

[7] Ko J. K. Y., Li R. H. W., Cheung V. Y. T. (2015). Caesarean scar pregnancy: a 10-year experience. *The Australian and New Zealand Journal of Obstetrics and Gynaecology*.

[8] Drever N., Bertolone J., Shawki M., Janssens S. (2020). Caesarean scar ectopic pregnancy: experience from an Australian tertiary centre. *The Australian and New Zealand Journal of Obstetrics and Gynaecology*.

[9] Deng J., Li S., Peng Y. (2020). Chinese herbal medicine for previous cesarean scar defect: a protocol for systematic review and meta-analysis. *Medicine*.

[10] Hung Y.-C., Kao C.-W., Lin C.-C. (2016). Chinese herbal products for female infertility in Taiwan: a population-based cohort study. *Medicine*.

[11] Vial Y., Petignat P., Hohlfeld P. (2000). Pregnancy in a cesarean scar. *Ultrasound in Obstetrics and Gynecology*.

[12] Bai J., Huang D. P., Li J. L. (2010). Color Doppler ultrasonography in the diagnosis and treatment of uterine scar pregnancy. *Chinese Journal of Medical Imaging Technology*.

[13] Ash A., Smith A., Maxwell D. (2007). Caesarean scar pregnancy. *BJOG: An International Journal of Obstetrics & Gynaecology*.

[14] Rotas M. A., Haberman S., Levgur M. (2006). Cesarean scar ectopic pregnancies: etiology, diagnosis, and management. *Obstetrics & Gynecology*.

[15] Bignardi T., Condous G. (2010). Transrectal ultrasound-guided surgical evacuation of Cesarean scar ectopic pregnancy. *Ultrasound in Obstetrics and Gynecology*.

[16] Xiao Z., Cheng D., Chen J., Yang J., Xu W., Xie Q. (2019). The effects of methotrexate and uterine arterial embolization in patients with cesarean scar pregnancy: a retrospective case–control study. *Medicine*.

[17] Shen L., Tan A., Zhu H., Guo C., Liu D., Huang W. (2012). Bilateral uterine artery chemoembolization with methotrexate for cesarean scar pregnancy. *American Journal of Obstetrics and Gynecology*.

[18] Wang G., Liu X., Wang D., Yang Q. (2015). Clinical analysis on selective uterine artery embolization combined with hysteroscopic surgery for exogenous cesarean scar pregnancy in 67 cases. *Zhonghua Fu Chan Ke Za Zhi*.

[19] Guex J.-J., Allaert F.-A., Gillet J.-L., Chleir F. (2006). Immediate and midterm complications of sclerotherapy: report of a prospective multicenter registry of 12,173 sclerotherapy sessions. *Dermatologic Surgery*.

[20] Chai Z.-Y., Yu L., Liu M.-M., Zhu T.-W., Qi F. (2018). Evaluation of the efficacy of ultrasound-guided local lauromacrogol injection combined with aspiration for cesarean scar pregnancy: a novel treatment. *Gynecologic and Obstetric Investigation*.

[21] Xu X. H., Liu G. R., He L. J., Wang Y. H., Zhao X. (2017). Effect and safety of ultrasound-guided injection of poly-alcohol on patients with scar pregnancy. *Chinese Journal of Biochemical Pharmaceutics*.

[22] Zhao Y., Zheng H.-X., Xu Y., Lin N. (2019). Estrogenic effect of the extract of qingyan formula on reproductive tissues in immature mice. *Evidence-Based Complementary and Alternative Medicine*.

[23] Kiyama R. (2017). Estrogenic potentials of traditional Chinese medicine. *The American Journal of Chinese Medicine*.

[24] Shao T. T. (2010). *Clinical Observation of Bushen Huoxue Tiaojing Decoction in the Treatment of Late Menstruation of Kidney Deficiency and Blood Stasis Type*.

[25] Kwon C.-Y., Lee B., Park K. S. (2018). Oriental herbal medicine and moxibustion for polycystic ovary syndrome: a meta-analysis. *Medicine*.

[26] Liu Y. L., Pan L. Z., Wang Y. (2018). Effects of the combined therapy of heat sensitive moxibustion and acupoint injection on endometrial receptivity of hypdrosalphinx infertility in the patients after hysteroscopy and laparoscopy. *Chinese Acupuncture & Moxibustion*.

[27] Weijing L., Wang A., Cao X., Chen J., Zhao S. (2012). Feature study on abdominal thermal infrared image in the treatment of dysmenorrhea by moxibustion at diji (SP 8). *Shanghai Journal of Acupuncture and Moxibustion*.

[28] Nelson P. L., Beck A., Cheng H. (2011). Transient receptor proteins illuminated: current views on TRPs and disease. *The Veterinary Journal*.

[29] Ye T.-j., Cheng H.-x. (2020). Therapeutic efficacy of moxibustion plus medicine in the treatment of infertility due to polycystic ovary syndrome and its effect on serum immune inflammatory factors. *Journal of Acupuncture and Tuina Science*.

